# Supplementary material for: Protein Crowding and Cholesterol Increase Cell Membrane Viscosity in a Temperature Dependent Manner
Source: J Chem Theory Comput. 2023 Apr 18;19(9):2630–43. doi: 10.1021/acs.jctc.3c00060 (PMC10173458; doi:10.1021/acs.jctc.3c00060)
Supplement: Supplementary file 1 — ct3c00060_si_001.pdf [file ct3c00060_si_001.pdf]

Supporting Information for: Protein Crowding and Cholesterol Increase Cell Membrane Viscosity in a Temperature Dependent Manner

Balázs Fábián,[*,†,‡] Ilpo Vattulainen,[¶] and Matti Javanainen[*,§,†,‖]

†*Institute of Organic Chemistry and Biochemistry of the Czech Academy of Sciences, CZ-16000 Prague 6, Czech Republic*

‡*Current address: Department of Theoretical Biophysics, Max Planck Institute of Biophysics, DE-60438, Frankfurt am Main, Germany*

¶*Department of Physics, University of Helsinki, FI-00560 Helsinki, Finland*

§*Institute of Biotechnology, University of Helsinki, FI-00790 Helsinki, Finland*

‖*Computational Physics Laboratory, Tampere University, FI-33720 Tampere, Finland*

E-mail: balazs.fabian@biophys.mpg.de; matti.javanainen@helsinki.fi

1 Supplementary Methods

1.1 All-Atom Simulations

We used the CHARMM36 lipid model for both phospholipids [1] and cholesterol. [2] The cholesterol-free membranes contained either 64, 256, or 1024 phospholipids, whereas in cholesterol-containing membranes, the cholesterol molecules were added without removing any phospholipids. The membranes were solvated (50 waters per lipid) by the CHARMMM-specific

TIP3P model.[3,4]

We used the simulation parameters recommended for CHARMM36 in GROMACS.[5] These simulation parameters, as well as the topologies, were obtained in GROMACS formats from CHARMM-GUI.[5] A time step of 2 fs was enabled by constraining bonds involving hydrogens by P-LINCS.[6,7] Calculation of non-bonded interactions was facilitated by buffered Verlet lists that tracked atomic neighbors.[8] For electrostatics, we used the smooth PME approach to include the long-range contributions.[9,10] Van der Waals and steric interactions were modeled by the Lennard-Jones potential with a cut-off of 1.2 nm, while the forces were switched to 0 between 1.0 and 1.2 nm to avoid discontinuity at the cut-off distance. The temperatures of the membrane and the solvent were separately coupled to the Nosé–Hoover thermostat[11,12] with a time constant of 1 ps. The target temperatures varied, as explained in the main text. The Parrinello–Rahman barostat[13] with a time constant of 5 ps, a compressibility of 4.5×10^{-5} bar^{-1}, and a reference pressure of 1 bar were used to maintain a constant pressure both in the plane of the membrane, as well as normal to it. All simulations were performed with the GROMACS 2020 package.[14,15]

The errors in viscosity were estimated as follows. For all-atom simulations, we first calculated the differences in lateral diffusion coefficients from the two bilayer leaflets and considered those as the error estimates for the diffusion coefficients. For CG simulations, we used previously reported error estimates in the works using these simulation data, Refs. 16 and 17. We then performed the relevant fit, either Eq. (2), (4), or (3) to the data, yet included error in each data point. These added errors were sampled from a normal distribution with a standard deviation corresponding to the error estimate for that data point. Such a fit was performed 10,000 times. The 10,000 membrane viscosity values were then binned in a histogram and fitted with a normal distribution whose standard deviation was used as the final error estimate. In some cases the distributions of membrane viscosities had long tails, and in these cases the values larger than 5 times the mean value were discarded as those likely correspond to a non-converged fit. For the fit of Eq. (3) to Set CG-1, we performed

the fit separately for each protein. Their mean value and standard error were then reported as the value and its error estimate, respectively. For cases for which we did not have well established error estimates, we chose to not report them in the main text.

2 Supplementary Results

2.1 Results From Coarse-Grained Simulations

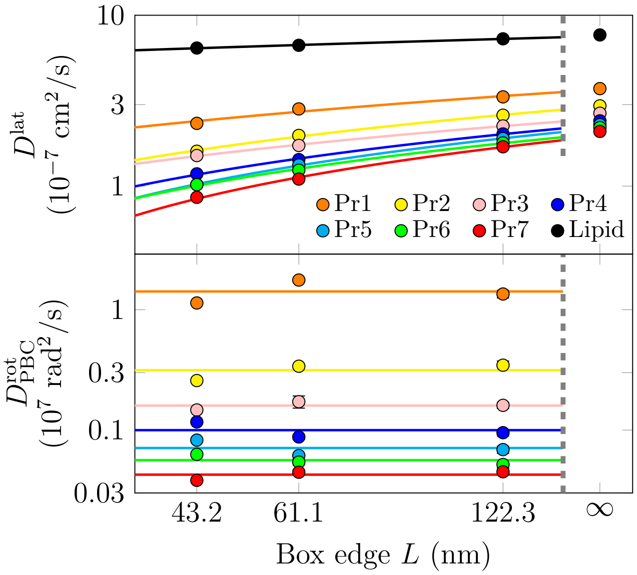

Figure S1: Dependence of lateral (top) and rotational (bottom) diffusion coefficients on membrane size extracted from the CG simulations where all proteins are present at once (Systems "P-" in Table 1, *i.e.* Set CG-1). For lateral diffusion, solid lines to show fits of Eq. (3) to the data. In addition to $D^{\text{lat}}_{\text{PBC}}$, we also plot the values at $L = \infty$ (D^{lat}_{∞}), extrapolated from Eq. 3. Importantly, the scaling relation also holds well for a lipid molecule, which experiences a very different environment due to inter-leaflet coupling. This independence of the correction on the type of studied molecule undergoing diffusive motion is a convenient feature of the hydrodynamic correction.[18] All shown figures for rotational diffusion are affected by PBCs, albeit the effect is minor for systems of this size.[19] We highlight this by fits of constant D^{rot}.

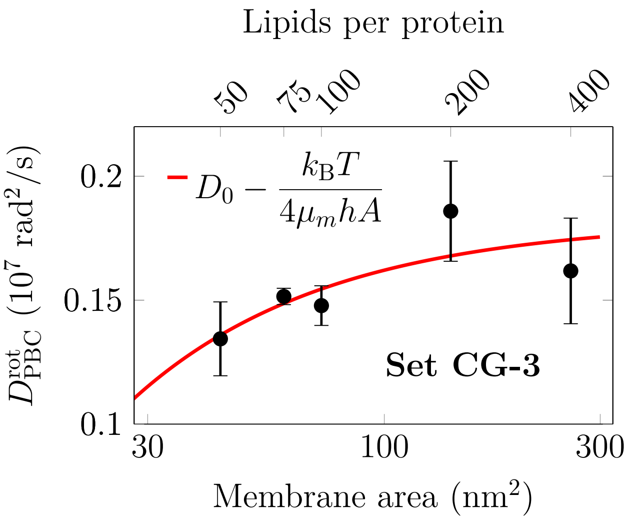

Figure S2: Dependence of rotational diffusion coefficients on membrane size in the CG simulations. Here, the medium-sized protein "P3" with an effective radius of 2.44 nm was used in a single-protein setting ("S-3-" in Table 1). The size dependence is satisfactorily captured by Eq. (4), and is hence in line with Ref. 19.

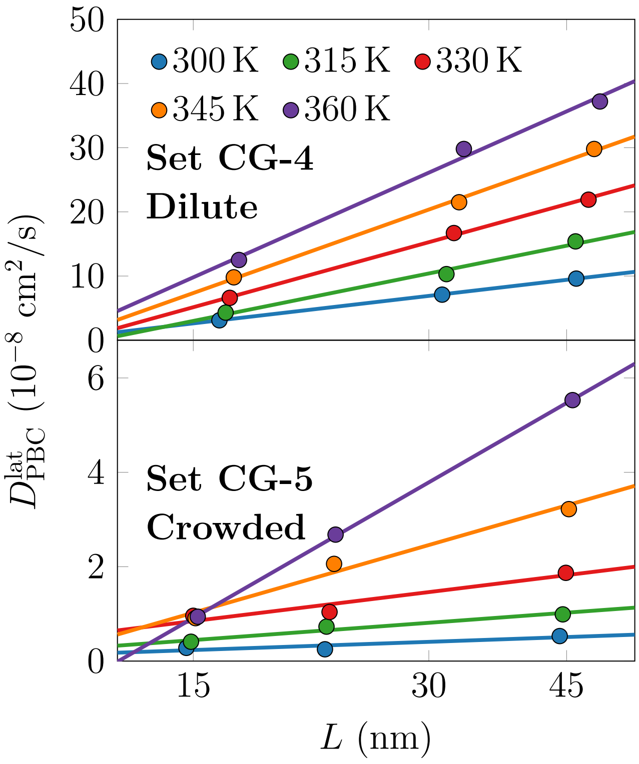

Figure S3: The dependence of lateral diffusion coefficients of proteins on the system size in dilute and crowded CG systems. The top panel shows data for dilute single-protein systems ("S-4^{400}", "S-4^{900},", and "S-4^{3600}", *i.e.* Set CG-4 in Table 1 in the main text), whereas the bottom panel shows data for crowded multi-protein systems ("M-4^{400}, M-4^{900}, and M-4^{3600}", *i.e.* Set CG-5 in Table 1 in the main text). The lines show fits of logarithmic dependence on the box size ($D^{\text{lat}} \sim \ln L$). Note the logarithmic scale on the abscissa.

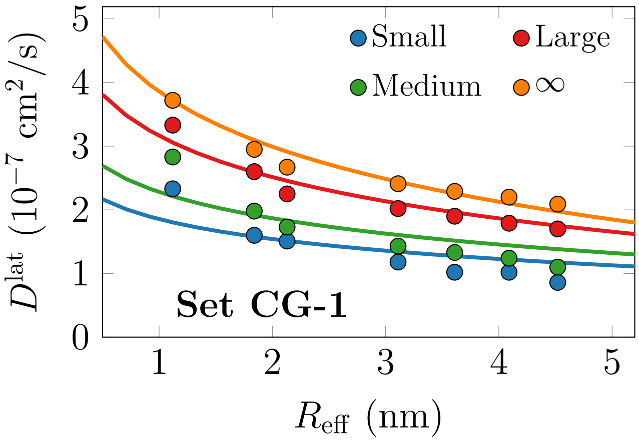

Figure S4: The dependence of lateral diffusion coefficients ($D^{\text{lat}}_{\text{PBC}}$) on the effective protein radius in the "P-" systems of three different sizes (Set CG-1), as well as in the infinite system size (D^{lat}_{∞}) extrapolated using Eq. (3) in the main text. These values are also shown in Fig. S1 as a function of system size. The solid lines show fits of the SD model, Eq. (1), to the data. Not surprisingly, only the diffusion coefficients of the infinite system size follow the SD-like $D^{\text{lat}} \propto \ln(R)$ dependence.

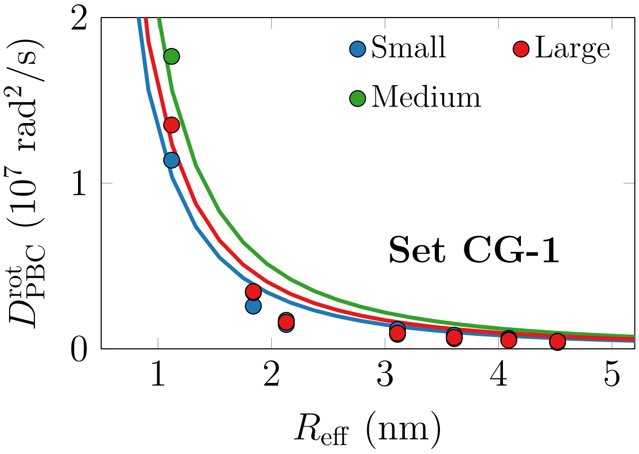

Figure S5: The dependence of rotational diffusion coefficients on the effective protein radius in the "P-" systems of three different sizes (Set CG-1). With these system sizes, the PBC-correction for rotational diffusion is minuscule (see also the bottom panel of Fig. S1). The solid lines show fits of the SD model for rotational diffusion, Eq. (2), to the simulation data.

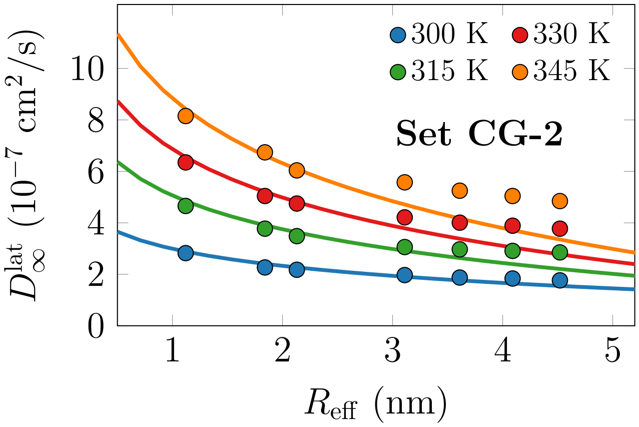

Figure S6: The dependence of lateral diffusion coefficients on the effective protein radius in the S-3 systems (of Set CG-2) at four different temperatures. All the shown values have been corrected for finite-size effects using the viscosity extracted from fits to finite-size dependency of lateral diffusion (Set CG-4 & Figs. S1 and 2) using Eq. (3). The solid lines show fits of the SD model for lateral diffusion, Eq. (1), to the data. The data measured at lower temperatures seem to better agree with the fits.

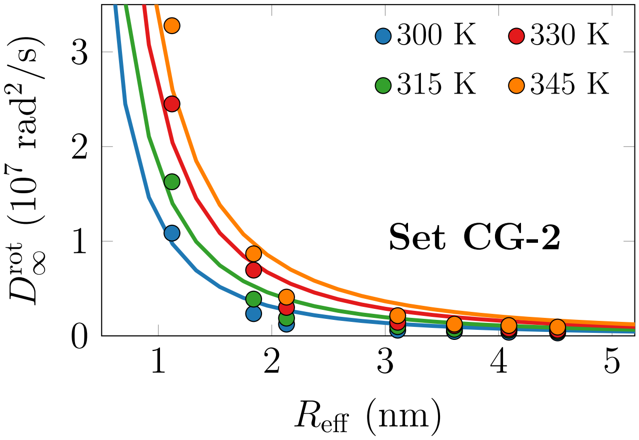

Figure S7: The dependence of rotational diffusion coefficients on the effective protein radius in the S-3 systems (of Set CG-2) at four different temperatures. All the shown values have been corrected for finite-size effects using the geometric correction factor of Eq. (4). The solid lines show fits of the SD model for rotational diffusion, Eq. (2), to the data.

2.2 Results From All-Atom Simulations

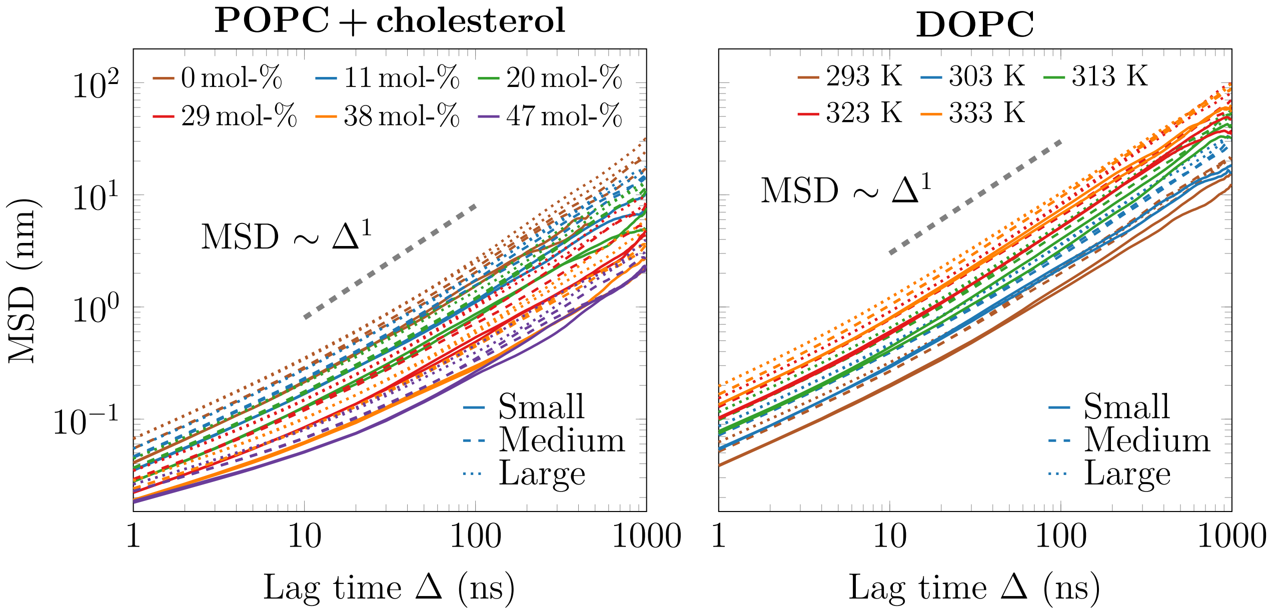

Figure S8: Examples of mean squared displacement (MSD) curves as a function of lag time Δ. Data are shown for the POPC/cholesterol mixtures at 298 K and for DOPC at different temperatures. The solid, dashed, and dotted lines show data for large, medium, and small membranes. For each system, two curves are shown, one for each leaflet. The dashed gray line highlights normal diffusion with MSD depending linearly on lag time.

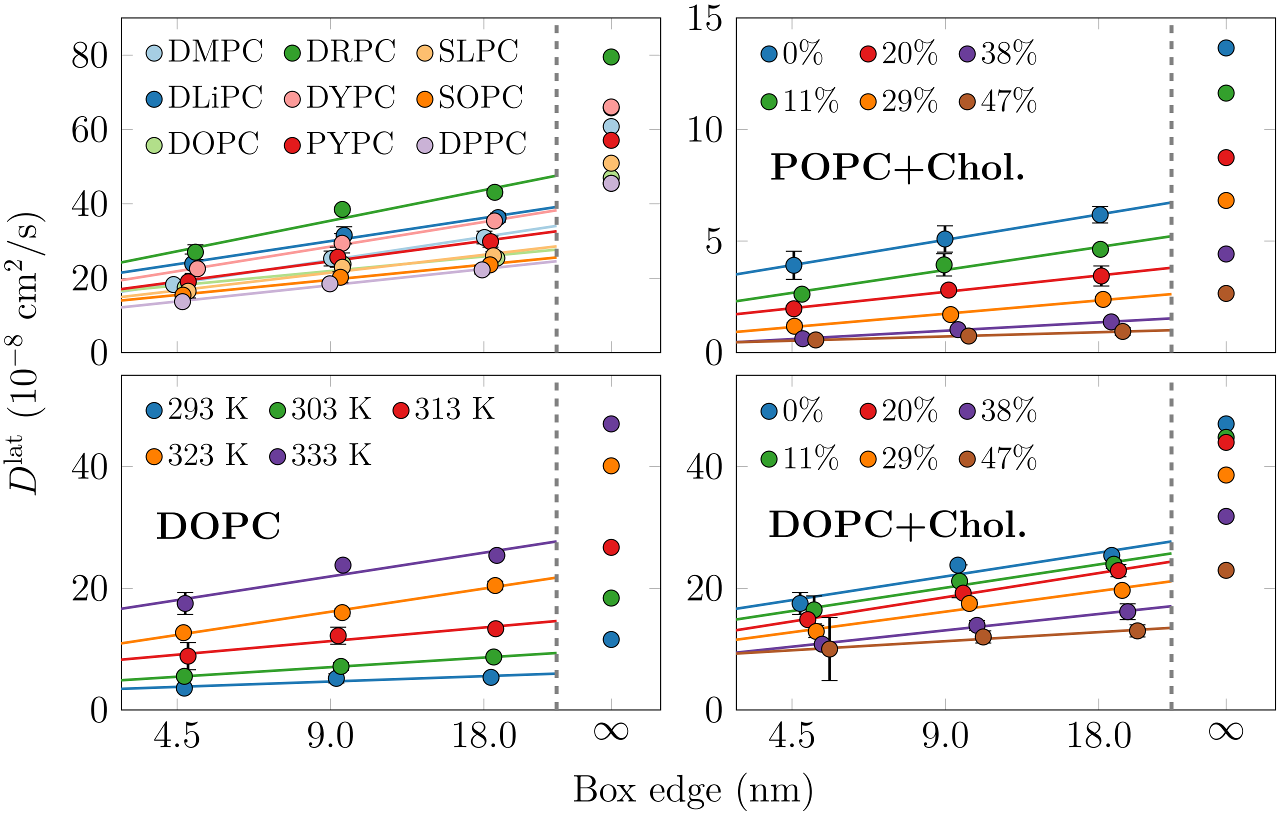

Figure S9: Lateral diffusion coefficients ($D^{\mathrm{lat}}_{\mathrm{PBC}}$) of lipids in all-atom simulations as a function of lateral size of the simulation box. The three markers show the values calculated from simulations performed using three different system sizes. The solid lines show fits of Eq. (3) to these three data points, and the marker at ∞ shows the value extrapolated to infinite box size from the fits ($D^{\mathrm{lat}}_{\infty}$). Data are shown for phosphatidylcholines with different acyl chains (top left panel) at 333 K, for DOPC at different temperatures (bottom left panel), for POPC mixed with cholesterol at 298 K, and for DOPC mixed with cholesterol at 333 K.

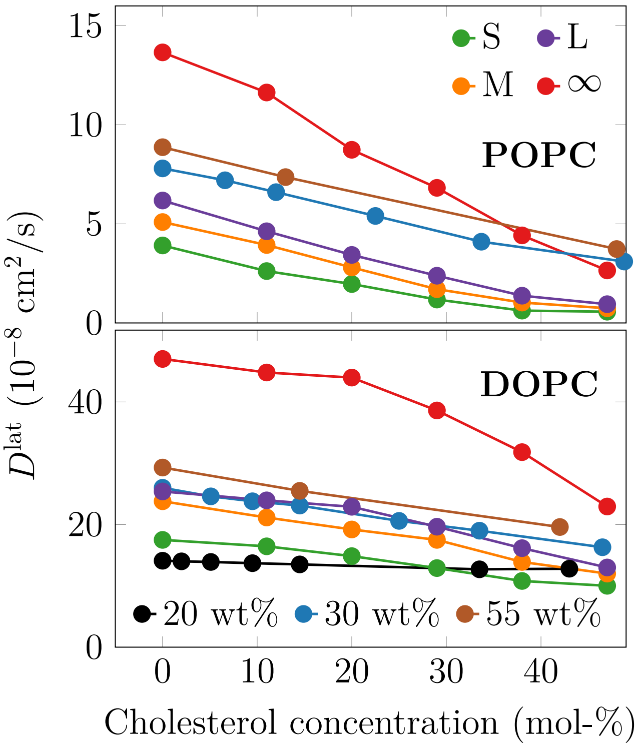

Figure S10: Lateral diffusion coefficients ($D^{\mathrm{lat}}_{\mathrm{PBC}}$) of POPC (298 K, top) and DOPC (333 K, bottom) as a function of cholesterol concentration, based on atomistic MD simulations. Values extracted from the simulation systems of three different sizes are shown; small (S), medium (M), and large (L). Moreover, values extrapolated to infinite ($D^{\mathrm{lat}}_{\infty}$) system sizes using Eq. (3) are shown. Experimental data have been measured using the pulsed field gradient NMR approach at multiple hydration levels (20, 30, and 55 weight% of water in the sample).[20,21] These values typically converge at the largest shown hydration level of 55 weight%.[21]

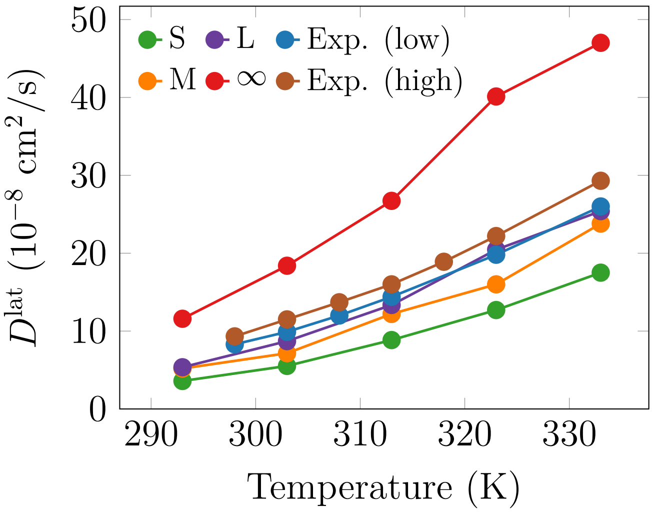

Figure S11: Lateral diffusion coefficients of DOPC ($D^{\mathrm{lat}}_{\mathrm{PBC}}$) as a function of temperature extracted from the atomistic simulation data of three different system sizes (small "S", medium "M", and large "L"), as well as from the data extrapolated to infinite system size ($D^{\mathrm{lat}}_{\infty}$) using Eq. (3) ($\infty$).

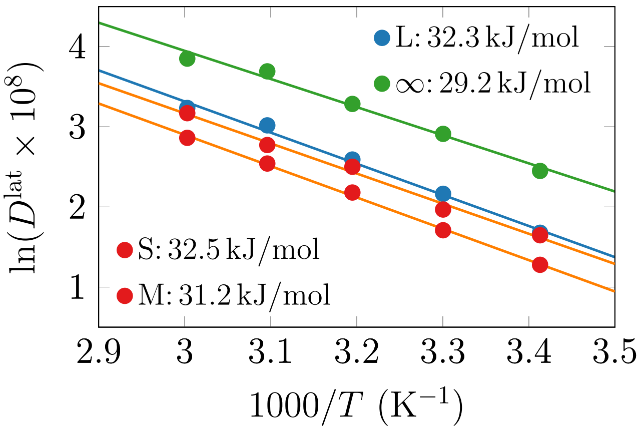

Figure S12: Lateral diffusion coefficients of DOPC shown in the Arrhenius description (Eq. (7)), *i.e.* the logarithm of the (unitless) diffusion coefficient is shown as a function of inverse temperature. Atomistic simulation sata are shown for three different system sizes ($D^{\mathrm{lat}}_{\mathrm{PBC}}$, small "S", medium "M", and large "L"), as well as for the extrapolated infinite system $D^{\mathrm{lat}}_{\infty}$) size using Eq. (3) ($\infty$). The activation energies extracted using the Arrhenius form are also shown in the legend labels.

References

(1) Klauda, J. B.; Venable, R. M.; Freites, J. A.; O'Connor, J. W.; Tobias, D. J.; Mondragon-Ramirez, C.; Vorobyov, I.; MacKerell Jr, A. D.; Pastor, R. W. Update of the CHARMM all-atom additive force field for lipids: validation on six lipid types. *J. Phys. Chem. B* **2010**, *114*, 7830–7843.

(2) Lim, J. B.; Rogaski, B.; Klauda, J. B. Update of the cholesterol force field parameters in CHARMM. *J. Phys. Chem. B* **2012**, *116*, 203–210.

(3) Jorgensen, W. L.; Chandrasekhar, J.; Madura, J. D.; Impey, R. W.; Klein, M. L. Comparison of simple potential functions for simulating liquid water. *J. Chem. Phys.* **1983**, *79*, 926–935.

(4) Durell, S. R.; Brooks, B. R.; Ben-Naim, A. Solvent-induced forces between two hydrophilic groups. *J. Phys. Chem.* **1994**, *98*, 2198–2202.

(5) Lee, J.; Cheng, X.; Swails, J. M.; Yeom, M. S.; Eastman, P. K.; Lemkul, J. A.; Wei, S.; Buckner, J.; Jeong, J. C.; Qi, Y., et al. CHARMM-GUI input generator for NAMD, GROMACS, AMBER, OpenMM, and CHARMM/OpenMM simulations using the CHARMM36 additive force field. *J. Chem. Theory Comput.* **2016**, *12*, 405–413.

(6) Hess, B.; Bekker, H.; Berendsen, H. J.; Fraaije, J. G. LINCS: a linear constraint solver for molecular simulations. *J. Comput. Chem.* **1997**, *18*, 1463–1472.

(7) Hess, B. P-LINCS: A parallel linear constraint solver for molecular simulation. *J. Chem. Theory Comput.* **2008**, *4*, 116–122.

(8) Páll, S.; Hess, B. A flexible algorithm for calculating pair interactions on SIMD architectures. *Comput. Phys. Commun.* **2013**, *184*, 2641–2650.

(9) Darden, T.; York, D.; Pedersen, L. Particle mesh Ewald: an N·log(N) method for Ewald sums in large systems. *J. Chem. Phys.* **1993**, *98*, 10089–10092.

(10) Essmann, U.; Perera, L.; Berkowitz, M. L.; Darden, T.; Lee, H.; Pedersen, L. G. A smooth particle mesh Ewald method. *J. Chem. Phys.* **1995**, *103*, 8577–8593.

(11) Nosé, S. A unified formulation of the constant temperature molecular dynamics methods. *J. Chem. Phys.* **1984**, *81*, 511–519.

(12) Hoover, W. G. Canonical dynamics: equilibrium phase-space distributions. *Phys. Rev. A* **1985**, *31*, 1695–1697.

(13) Parrinello, M.; Rahman, A. Polymorphic transitions in single crystals: a new molecular dynamics method. *J. Appl. Phys.* **1981**, *52*, 7182–7190.

(14) Abraham, M. J.; Murtola, T.; Schulz, R.; Páll, S.; Smith, J. C.; Hess, B.; Lindahl, E. GROMACS: high performance molecular simulations through multi-level parallelism from laptops to supercomputers. *SoftwareX* **2015**, *1*, 19–25.

(15) Páll, S.; Zhmurov, A.; Bauer, P.; Abraham, M.; Lundborg, M.; Gray, A.; Hess, B.; Lindahl, E. Heterogeneous parallelization and acceleration of molecular dynamics simulations in GROMACS. *J. Chem. Phys.* **2020**, *153*, 134110.

(16) Javanainen, M.; Martinez-Seara, H.; Metzler, R.; Vattulainen, I. Diffusion of integral membrane proteins in protein-rich membranes. *J. Phys. Chem. Lett.* **2017**, *8*, 4308–4313.

(17) Javanainen, M.; Ollila, O. S.; Martinez-Seara, H. Rotational diffusion of membrane proteins in crowded membranes. *J. Phys. Chem. B* **2020**, *124*, 2994–3001.

(18) Vögele, M.; Köfinger, J.; Hummer, G. Hydrodynamics of diffusion in lipid membrane simulations. *Phys. Rev. Lett.* **2018**, *120*, 268104.

(19) Vögele, M.; Koefinger, J.; Hummer, G. Finite-size corrected rotational diffusion coefficients of membrane proteins and carbon nanotubes from molecular dynamics simulations. *J. Phys. Chem. B* **2019**,

(20) Filippov, A.; Orädd, G.; Lindblom, G. The effect of cholesterol on the lateral diffusion of phospholipids in oriented bilayers. *Biophys. J.* **2003**, *84*, 3079–3086.

(21) Filippov, A.; Orädd, G.; Lindblom, G. Influence of cholesterol and water content on phospholipid lateral diffusion in bilayers. *Langmuir* **2003**, *19*, 6397–6400.
